# Rv3722c Promotes *Mycobacterium tuberculosis* Survival in Macrophages by Interacting With TRAF3

**DOI:** 10.3389/fcimb.2021.627798

**Published:** 2021-02-25

**Authors:** Yingying Lei, Xiaojian Cao, Weize Xu, Bing Yang, Yangyang Xu, Wei Zhou, Shuang Dong, Qijun Wu, Khaista Rahman, Rohit Tyagi, Shuhong Zhao, Xi Chen, Gang Cao

**Affiliations:** ^1^ State Key Laboratory of Agricultural Microbiology, Huazhong Agricultural University, Wuhan, China; ^2^ College of Veterinary Medicine, Huazhong Agricultural University, Wuhan, China; ^3^ Key Laboratory of Agricultural Animal Genetics, Breeding, Reproduction of Ministry of Education, Huazhong Agricultural University, Wuhan, China; ^4^ The Cooperative Innovation Center for Sustainable Pig Production, Huazhong Agricultural University, Wuhan, China; ^5^ College of Animal Science and Technology, Huazhong Agricultural University, Wuhan, China; ^6^ Bio-Medical Center, Huazhong Agricultural University, Wuhan, China; ^7^ Key Laboratory of Development of Veterinary Diagnostic Products, Ministry of Agriculture, Huazhong Agricultural University, Wuhan, China

**Keywords:** *Mycobacterium tuberculosis*, Rv3722c, TRAF3, cytokines, intracellular survival

## Abstract

*Mycobacterium tuberculosis* (*M.tb*) secretes numerous proteins to interfere with host immune response for its long-term survival. As one of the top abundant *M.tb* secreted proteins, Rv3722c was found to be essential for bacilli growth. However, it remains elusive how this protein interferes with the host immune response and regulates *M.tb* survival. Here, we confirmed that Rv3722c interacted with host TRAF3 to promote *M.tb* replication in macrophages. Knock-down of TRAF3 attenuated the effect of Rv3722c on the intracellular *M.tb* survival. The interaction between Rv3722c and TRAF3 hampered MAPK and NF-κB pathways, resulting in a significant increase of IFN-β expression and decrease of IL-1β, IL-6, IL-12p40, and TNF-α expression. Our study revealed that Rv3722c interacted with TRAF3 and interrupted its downstream pathways to promote *M.tb* survival in macrophages. These findings facilitate further understanding of the mechanism of *M.tb* secreted proteins in regulating the host cell immune response and promoting its intracellular survival.

## Introduction

Tuberculosis, caused by the bacillus *Mycobacterium tuberculosis* (*M.tb*), remains the leading cause of morbidity and mortality from a single infectious pathogen throughout the world. An estimated 10.0 million people fell ill with tuberculosis and around 1.4 million deaths worldwide in 2019 (www.who.int). As an aerosol transmitted pathogen, *M.tb* is firstly recognized by alveolar macrophages and persists in infected macrophages. The infection can trigger a wide range of host cells immune defenses, such as inflammation, phagocytosis, autophagy, and apoptosis, to eliminate invaded *M.tb* (Lerner et al., 2015). However, *M.tb* has developed numerous strategies to escape from host immune clearance for long-term persistence (Hmama et al., 2015; Liu et al., 2017). During infection, *M.tb* secretes a variety of proteins to interrupt the immune response in host cells (Wang J. et al., 2015; Wang et al., 2017; Stamm et al., 2019). Thus, elucidation of secreted protein functions can facilitate understanding of the pathogenesis of tuberculosis.

It has been well demonstrated that PtpA, an *M.tb* secreted tyrosine phosphatase, suppresses host innate immunity by dephosphorylating c-Jun N-terminal kinase (JNK) and p38 (Wang J. et al., 2015). MPT53, an *M.tb* secreted disulfide-bond-forming-like protein, interacts with TAK1 in a TLR2- or MyD88-independent manner, and induces the host inflammatory responses (Wang et al., 2019). Rv3722c, an *M.tb* secreted antigen, is annotated as an aspartate aminotransferase, which is a transcription factor that belongs to the GntR family (Grishin et al., 1995; Bramucci et al., 2011; Ortega et al., 2016; Penn et al., 2018; El-Gebali et al., 2019; Yang et al., 2019; Jansen et al., 2020). Proteomics analysis revealed that Rv3722c belongs to the top 10–25% of the most abundant proteins in *M.tb* (Wang M, et al., 2015). Rv3722c was identified to be essential for bacillus growth *in vitro* and infection (Griffin et al., 2011; Jansen et al., 2020). Previously, we identified that Rv3722c can interact with host immune-related proteins in different pathways (Yang et al., 2018), suggesting that Rv3722c may play an important role in disturbing host immune clearance. However, it remains largely uncharacterized how Rv3722c interplays with host targets and to turn host immune responses.

During infection, multiple conserved pathogen-associated-molecular patterns (PAMPs) of *M.tb* are recognized by a variety of phagocytic pattern recognition receptors (PRRs), which plays a crucial role in the initiation of signaling cascades and subsequently promotes cytokines and chemokines production in macrophages (Killick et al., 2013; Stamm et al., 2015). For example, ESAT-6 (Rv3875) directly interacts with TLR2 and inhibits activation of nuclear factor-κB (NF-κB) and IRFs in macrophages (Pathak et al., 2007). Following recognition by TLR2, secreted *M.tb* lipoprotein MPT83 (Rv2873) activates mitogen-activated protein kinase (MAPK) signaling cascades and subsequently induces the production of cytokines, including TNF-α, IL-6, and IL-12p40 in macrophages (Wang et al., 2017). TNF receptor-associated factor 3 (TRAF3) is a tri-faced immune regulator that negatively regulates the NF-κB and MAPK, but positively controls the production of type I interferon (Häcker et al., 2011). TRAF3 plays an essential role in cytokine production during host innate immune response to microbe infection. Among which, interleukin-1α (IL-1α), interleukin-1β (IL-1β), tumor necrosis factor-alpha (TNF-α), IL-12 family, IL-6, and IL-10 are the major cytokines in defense against *M.tb* infection (Guirado et al., 2013).

In the present study, we confirmed the host protein TRAF3 interacts with *M.tb* secreted protein Rv3722c by using yeast two-hybrid (Y2H), bimolecular fluorescence complementation (BiFC) and co-immunoprecipitation (Co-IP) approaches. The biological roles of the interaction between TRAF3 and Rv3722c were further investigated. Collectively, we proposed a model in which Rv3722c and TRAF3 interaction promotes *M.tb* replication in macrophages by affecting MAPK and NF-κB mediated cytokine expression.

## Materials and Methods

### Bacterial Culture and Infection

*M.tb* strain H37Ra (ATCC 25177) was cultured in Middlebrook 7H9 broth (BD PharMingen, USA) supplemented with 0.5% glycerol, 0.05% Tween-80, and 10% oleic acid albumin dextrose catalase (OADC, BD PharMingen, USA). For confocal microscopy, *M.tb* were transformed with a plasmid expressing RFP and maintained by addition of hygromycin B (50 μg/ml). For macrophages infection, the bacterial culture optical density at 600 nm (OD_600nm_) were adjusted to achieve the required multiplicity of infection (MOI) and centrifuged at 3,000 g for 10 min to pellet the bacteria. The pellet was resuspended in infection medium and passed several times through an insulin syringe to disperse the bacteria. In addition, 50 μl from serially diluted inoculate were plated to Middlebrook 7H11 agar (BD PharMingen, USA) plate and then incubated at 37°C to count the number of viable bacteria (colony-forming units—CFUs).

### Plasmids Construction

Rv3722c was amplified and cloned into yeast expression vector pmBD, eukaryotic expression vector pcDNA3.1-V5/hisB or bjun-KN151, and lentiviral expression vector pHKO-eGFP to obtain pmBD-Rv3722c, pcDNA3.1-V5-Rv3722c, ΔJun-Rv3722c, or pHKO-eGFP-Rv3722c, respectively. TRAF3 was amplified and cloned into yeast expression vector pmAD, eukaryotic expression vector pCAGGS-HA and bFos-KN151 to obtain pmAD-TRAF3, pCAGGS-HA-TRAF3, or ΔFos-TRAF3, respectively. All insertions were confirmed by Sanger sequencing (Shanghai Sangon Biotech, China).

### Cell Culture and Transient Transfection

HEK 293T (ATCC CRL-3216™) and RAW264.7 (ATCC TIB-71™) cells were grown in Dulbecco’s modified Eagle’s medium (DMEM) containing 10% fetal bovine serum (FBS), 10 U/ml penicillin, and 10 mg/ml streptomycin at 37°C with 5% CO_2_. Before transfection, 5 × 10^5^ of HEK 293T or RAW264.7 cells were seeded into six-well plates and grown to 80–90% confluence. Then 1 μg of recombinant plasmids was transfected into HEK 293T cells using calcium phosphate precipitation or into RAW264.7 cells using Lipofectamine 2000 reagent (Invitrogen).

### Intracellular Bacterial Viability Assay

Then 5.0 × 10^4^ cells per well were seeded in 24-well plates per well for 24 h and were incubated with *M.tb* (MOI = 10) for 6 h at 37°C in 5% CO_2_. The cells were washed three times with pre-warmed PBS to remove extracellular *M.tb*, and supplied with fresh medium with 5% FBS containing amikacin (50 μg/ml) (referred to as day 0). The medium was changed every 2 days. The infected cells were lysed at indicated time points using 0.5 ml of sterile 0.1% Tween-80 in water, and viable *M.tb* were enumerated by serial dilution of lysates and plating as described above. The total number of viable intracellular bacteria would be calculated as the following formula: total CFUs per ml/well = CFU × dilution × 20.

### Antibodies and Reagents

Antibodies used in the current study were anti: HA-tag mAb-Magnetic Agarose (MBL, M180-10), HA tag (proteintech, 51064), V5 tag (proteintech, 14440), NF-κB p65 (Cell Signaling Technology, 8242), Phospho-NF-κB p65 (Cell Signaling Technology, 3033), MAPK family antibody sample kit (Cell Signaling Technology, 9926), Phospho-MAPK family antibody sampler kit (Cell Signaling Technology, 9910), TRAF3 (Cell Signaling Technology, 4729), NF-κB2 p100/p52 (Cell Signaling Technology, 4882), Alexa Fluor^®^ 488 Goat anti-mouse IgG (Invitrogen, A-11001), DAPI (Beyotime, C1002), Proteinase inhibitor cocktail (Sigma, P8340), Phosphatase inhibitor cocktail 3 (Sigma, P0044).

### Yeast Two-Hybrid

Full-length of Rv3722c and TRAF3 were restriction cloned into pmBD and pmAD plasmids of the GAL4 Y2H system (Clontech, Mountain-view, CA, USA) respectively as described (Yang et al., 2018). pmAD-TRAF3 was transformed into yeast strain Y187, and plated on SD/-T selective plates. pmBD-Rv3722c was transformed into yeast strain GoldY2H, and plated on SD/-L selective plates. GoldY2H cells harboring Rv3722c plasmid and Y187 cells harboring TRAF3 plasmid were hybridized and selected on SD/Leu^−^Trp^−^His^−^Ade^−^ selective plates. The yeast strains were transformed with the respective constructs and transformants were selected on minimal media lacking leucine and tryptophan (–LT). Interactions were assessed by growing trans-formants in liquid culture at 30^°^C and spotting on SD/Leu^−^Trp^−^His^−^Ade^−^ selective plates. Plates were imaged after 3–5 d growth at 30^°^C.

### Immunoprecipitation and Immunoblotting

HEK 293T cells were transiently co-transfected with pcDNA3.1-V5-Rv3722c and pCAGGS-HA-TRAF3. After 36 h, cells were washed with PBS and then lysed in cell-lysis buffer (50 mM Tris-HCl, pH 7.5, 150 mM NaCl, 0.05% Nonidet P-40) for western blotting and immunoprecipitation, supplemented with 1% protease inhibitor cocktail (Sigma, P8340). After incubation for 30 min on ice, whole cell lysates were centrifuged at 10,000 g for 10 min at 4°C to remove debris. The cell lysates were incubated with HA-tag mAb-Magnetic Agarose beads (MBL, M180-10) for 4 h at 4°C. The immunocomplexes samples were centrifuged, washed three times with cell-lysis buffer, and boiled with SDS loading buffer for 5–10 min. After separation by 12% SDS-PAGE, equivalent amounts of protein were electroblotted onto polyvinylidene difluoride membranes (Millipore, Bedford, MA, USA). The membrane was blocked with Tris-buffered saline containing 0.05% Tween 20 (TBST) and 5% fat-free dry milk for 2 h at room temperature and then incubated overnight with primary antibodies. After washing three times with TBST, the membranes were further incubated for 1 h at room temperature with corresponding horseradish peroxidase-conjugated secondary antibody in appropriate dilution. The immunoreactive protein bands were visualized by clarity western ECL substrate (Bio-Rad, 1705060).

### Protein-Protein Docking Simulation

To determine the structural basis of *M.tb* R3722c bindings to TRAF3, we performed a protein-protein docking. The structures of Rv3722c in complex with TRAF3 were determined by Discovery Studio 2018 software based on the Macromolecules properties. The X-ray crystal structures of Rv3722c (PDB code: 5c6u) and TRAF3 (PDB code: 4ghu) were downloaded from RCSB Protein Data Bank (https://www.rcsb.org). The binding patterns of protein-protein were predicted by ZDOCK modulate based on Dock and Analyze Protein Complexes in Macromolecules properties according to tutorials of the software. TRAF3 and Rv3722c were defined as receptor protein and ligand protein respectively. The predicted protein complexes were optimized by RDOCK modulate. Docking results were analyzed and visualized by Analyze Protein Interface modulate in macromolecules properties.

### Acquisition of Lentivirus Containing Target Genes and RAW264.7 Cell Line Construction

shRNAs targeting TRAF3 (shTRAF3-1, shTRAF3-2, and shTRAF3-3) were designed using BLOCK-iT™ RNAi Designer (Invitrogen) (shown in **Table S1**). Rv3722c and shTRAF3 were inserted into the lentiviral vector pHKO-eGFP-puro and pLKO-eGFP-puro respectively. To generate lentiviruses expressing Rv3722c, HEK293T cells were seeded into six-well plates and co-transfected with 1 μg of pMD.2G, 2 μg of pSPAX, and 2 μg of pHKO-Rv3722c or pLKO-shTRAF3 per well, pHKO-eGFP-puro vector or pLKO-NC-puro was used as a negative control. At 72 h post-transfection, the cell culture supernatants with the virus were collected and filtered with a 0.45 μm filter. Viruses were pelleted by centrifuging 30 min at 5,000 g after incubating with virus concentration solution for overnight. Viral pellets were resuspended, mixed with polybrene, and incubated with RAW264.7 cells. After 24 h incubation, the medium was replaced with fresh medium containing 10% FBS and 4 μg/ml puromycin (Selleck, S7417) to purify cell lines. qRT-PCR was performed to detect overexpression or knockdown efficiency of target genes.

### RNA Extraction, RT-PCR and Quantitative Real-Time PCR (qRT-PCR) Analysis

Total RNAs were isolated using Trizol reagent (Invitrogen, 15596026) according to the manufacturer’s instructions. One µg of total RNAs were processed directly to cDNA with HiScript III 1^st^ strand cDNA synthesis kit (Vazyme, R312), following the manufacturer’s instructions. qRT-PCR reactions were performed in a 20 µl volume of qPCR SYBR Green Master Mix (Thermo Fisher, A25742). All of the reactions were triplicated and performed in the ABI 7300 system (Applied Biosystems). To confirm the specificity of amplification, the PCR products from each primer pair were subjected to a melting curve analysis and electrophoresis in 2% agarose gel. Primers used for qRT-PCR were in **Table S2**. Further, the Ct values for each gene amplification were normalized with internal control β-actin by the 2^–ΔΔCt^ method. All of the qPCR experiments were conducted in duplicates in each experiment, and experiments were replicated at least three times.

### Fluorescence and Confocal Microscopy

For fluorescent microscopy, 5.0 × 10^4^ HEK 293T cells per well were seeded in a 24-well plate for 24 h and were transiently transfected with ΔFos-TRAF3 or ΔJun-Rv3722c for 36 h at 37°C in 5% CO_2_, ΔFos or ΔJun vector was used as a negative control.

For confocal microscopy, 5.0 × 10^4^ RAW264.7 cells per well were seeded onto cover slips in a 24-well plate for 24 h and infected with RFP-H37Ra (MOI = 10) for 6 h at 37°C in 5% CO_2_. The infected cells were fixed with 4% paraformaldehyde overnight at indicated time points. Specimens were incubated with DAPI (1:5,000) for 5 min and then mounted onto microscope slides using a prolong antifade reagent with DAPI (Invitrogen). Images were obtained with an Olympus confocal laser microscope system equipped with FV10 ASW Imaging Software (Version 4.2, Olympus). The number of bacteria from 100 transfected positive cells was analyzed. All infections were performed in triplicate.

### NGS Library Preparation and Sequencing

Total RNAs were isolated from RAW-Vector and RAW-Rv3722c cells, and the mRNA-seq library was constructed by VAHTS Universal V6 RNA-seq Library Prep Kit for MGI (Vazyme, NRM604) following the manufacture’s instruction. The prepared library was sequenced on MGISEQ-2000 (BGI, China) platform.

### NGS Data Analysis and Visualization

The protein-protein-interaction (PPI) sub-network between Rv3722c and host immune-related proteins was extracted from the entire PPI network (Yang et al., 2018).

Mouse hisat2 index was downloaded from (ftp://ftp.ccb.jhu.edu/pub/infphilo/hisat2/data/grcm38.tar.gz). Gene expression level list was exported by the protocol provided by Pertea et al. (2016), and gene count list corresponding to every sample were merged and differential analyzed by deseq2 (Love et al., 2014). The volcano plot was generated by R package ggplot2 (https://ggplot2.tidyverse.org) and ggrepel (https://CRAN.R-project.org/package=ggrepel). KEGG enrichment was conducted by R package cluster Profiler (Yu et al., 2012). Heatmap was generated by R package pheatmap (https://CRAN.R-project.org/package=pheatmap), a polarized heatmap was plotted by Tbtools (Chen et al., 2020).

### Data Availability

The sequencing data of PPIs between *M.tb* and host was downloaded from Gene Expression Omnibus (GEO) under accession number GSE93036.

RNA-sequencing data has been deposited in GEO under accession number GSE157419, and is publicly available on January 1^st^, 2021.

### Statistical Analysis

Numerical data were analyzed and plotted by using GraphPad Prism 7.0 (La Jolla, CA, USA) software from three independent experiments shown as mean ± SD or SEM. Evaluation of the significance of differences between groups was performed by using two-way ANOVA or student *t*-test. Statistical difference was considered to be significant when *p* < 0.05 and the *p* values of < 0.05, < 0.01, < 0.001 were respectively indicated as *, **, and *** in figures.õ

## Results

### Rv3722c Interacts With TRAF3

**Figure 1:**
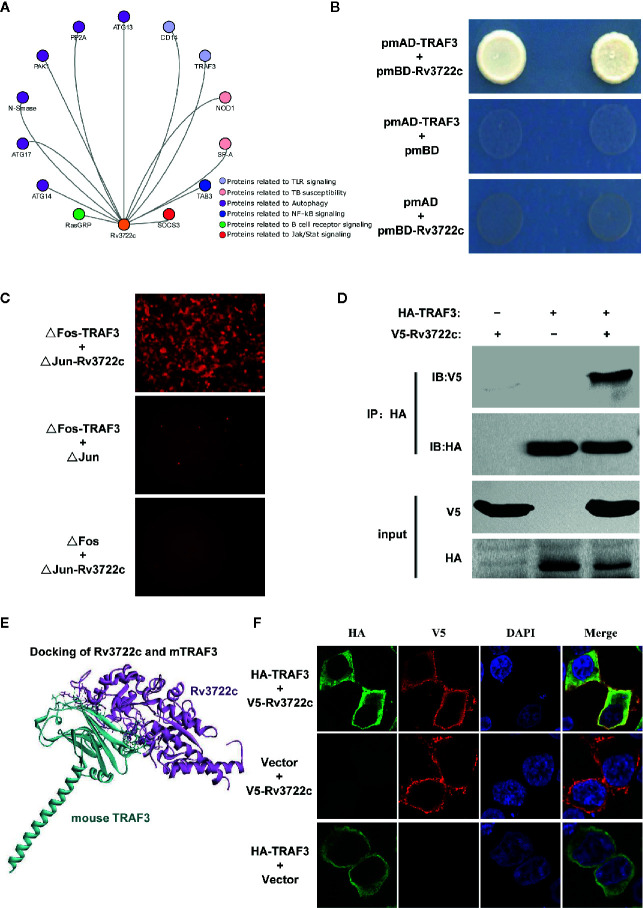
Rv3722c interacts with TRAF3. **(A)** Delineation of the filtered PPI sub-network between Rv3722c and host proteins. The orange node represented Rv3722c and other colors represented host different immune pathway proteins. **(B)** Verification of the protein-protein interaction by Y2H assay. Rv3722c in the pmBD destination vector was used as bait. TRAF3 in the pmAD destination vector was used as prey. Co-transformation of pmBD empty vector with pmAD vector containing TRAF3 and co-transformation of the pmAD empty vector with pmBD vector containing Rv3722c were used as negative controls. Yeast cells grown on SD/Leu^−^Trp^−^His^−^Ade^−^ selective plates indicated the interaction between Rv3722c and TRAF3. **(C)** Verification of the protein-protein interaction by BiFC assay. ΔJun-Rv3722c was co-transfected with ΔFos-TRAF3 and the negative controls into HEK 293T cells, respectively. Cells were harvested 36 h after transfection for fluorescence microscopy-based image analysis. The interaction between Rv3722c and TRAF3 allowed the re-formation of a bimolecular fluorescent complex. **(D)** Verification of the protein-protein interaction by Co-IP assay. V5-tagged Rv3722c and HA-tagged TRAF3 were co-transfected into HEK 293T cells. Cells were harvested 36 h after transfection for Co-IP assay. Co-transfection of V5-tagged Rv3722c with HA-tagged empty vector and HA-tagged TRAF3 with V5-tagged empty vector were used as negative controls. **(E)** Protein-protein docking simulated the interaction of Rv3722c and TRAF3. The purple structure represented Rv3722c and the blue-green structure represented TRAF3. **(F)** Verification of the protein-protein co-localized by immunofluorescence confocal microscopy assay. V5-tagged Rv3722c and HA-tagged TRAF3 were co-transfected into HEK 293T cells. Cells were harvested at 36 h after transfection for immunofluorescence confocal microscopy assay. Co-transfection of V5-tagged Rv3722c with HA-tagged empty vector and HA-tagged TRAF3 with V5-tagged empty vector were used as negative controls. V5-tagged Rv3722c and HA-tagged TRAF3 signals were co-localized in the cytoplasm of HEK 293T cells. All experiments were performed in triplicate.

It has been shown that aspartate aminotransferase R3722c mediates an essential role in metabolism and is required for virulence of *M.tb* (Jansen et al., 2020). Previously, we constructed a filtered PPI network with 441 PPIs between *M.tb* membrane and secreted proteins and host immunity-related proteins (Yang et al., 2018). To investigate the interaction of Rv3722c and host proteins, a filtered PPI network containing 13 PPIs of Rv3722c with host proteins was sorted out (**Figure 1A**). As the RLL-Y2H screening system might generate false-positive data, the 13 PPIs were then subjected to interaction retest by using Y2H, BiFC and Co-IP approach. Of note, we identified TRAF3, an important multifunctional immune regulator involved in MAPK and NF-κB pathways as an interaction target of Rv3722c. As shown in **Figure 1B**, the interaction between Rv3722c and TRAF3 was confirmed by using the Y2H experiment. To further validate this interaction by other independent approaches, BiFC and Co-IP were performed. As shown in **Figure 1C**, the BiFC assay showed strong fluorescent signals when co-transfection of ΔFos-TRAF3 with ΔJun-Rv3722c into HEK 293T cells, neither ΔFos-TRAF3 with ΔJun vector control nor ΔFos vector control with ΔJun-Rv3722c. In addition, the specific interaction between Rv3722c and TRAF3 was verified by a Co-IP assay (**Figure 1D**).

Next, we obtained the protein structure of Rv3722c and TRAF3 from the RCSB Protein Data Bank (https://www.rcsb.org) and predicted the possible interaction confirmation by protein-protein docking simulation. **Figure 1E** showed the structure of Rv3722c and TRAF3 complex, which demonstrated the possibility of the interaction between the two proteins. In detail, 16 amino acids from TRAF3 were in contact with 16 residues of Rv3722c, which form 14 hydrogen bonds, 10 hydrophobics, and 3 electrostatics. Based on these findings, we then hypothesized that Rv3722c and TRAF3 would be co-localized in cells. To this end, V5-tagged Rv3722c and HA-tagged TRAF3 were co-transfected into HEK 293T cells and subjected to immunofluorescence confocal microscopic analysis. As shown in **Figure 1F**, Rv3722c and TRAF3 were indeed co-localized in the cytoplasm of transfected HEK 293T cells, providing the possibility of the interaction between Rv3722c and TRAF3 in the same subcellular location. Together, these data unambiguously showed that *M.tb* protein Rv3722c interacts with host protein TRAF3, suggesting that Rv3722c may participate in the regulation of host immune response for its survival.

### Rv3722c Promotes *M.tb* Survival in Murine Macrophages

**Figure 2:**
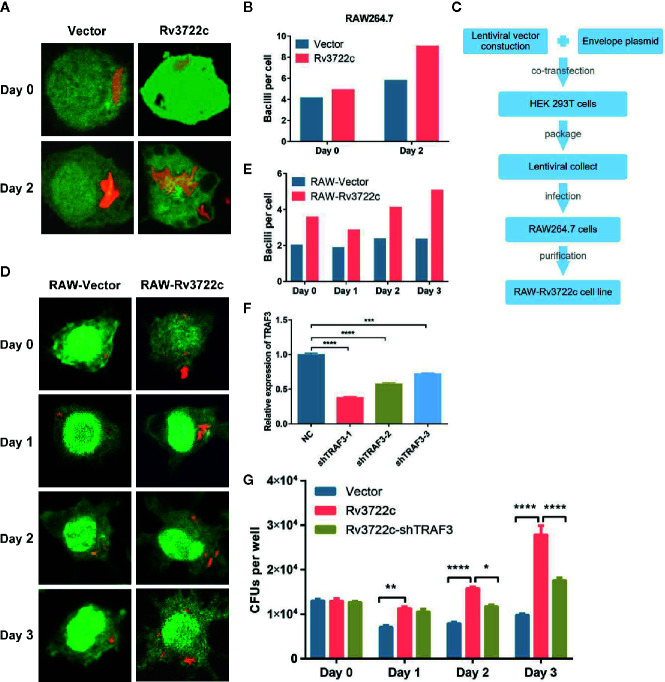
Knock-down of TRAF3 attenuates the effect of Rv3722c on *M.tb* proliferation. **(A)** pcDNA3.1-V5-Rv3722c or pcDNA3.1-V5 vector control and pAAV-eGFP were transiently co-transfected into RAW264.7 cells. Cells were infected with RFP-H37Ra at an MOI of 10 after transfected for 6 h. Cells were harvested for confocal microscopy-based image analysis at the indicated times. **(B)** Quantification of the average number of intracellular bacilli per cell in a total of 100 cells. **(C)** Flowchart of RAW-Rv3722c cell line construction. **(D)** RAW-Vector and RAW-Rv3722c cell lines were infected with RFP-H37Ra at an MOI of 10. Cells were harvested for confocal fluorescence microscopy-based image analysis at the indicated times. **(E)** Quantification of the average number of intracellular bacilli per cell in a total of 100 cells. **(F)** Identification of the mRNA expression of endogenous TRFA3 by qRT-PCR. **(G)** After infection of RAW-Rv3722c cells with lentivirus containing shTRAF3-1 gene for 48 h, the cells were infected with H37Ra at an MOI of 10. Cells were lysed and the CFUs of intracellular *M.tb* were detected by plating on 7H11 plates at the indicated times. Data shown in **(F, G)** are mean ± SD of three independent experiments. *P < 0.05; **P < 0.01; ***P < 0.001; ****P < 0.0001; ns, not significant; by two-way ANOVA or student *t*-test. All experiments were performed in triplicate.

As Rv3722c is essential for *M.tb* growth *in vitro* and interacts with host proteins such as TRAF3 (Yang et al., 2018; Jansen et al., 2020), we hypothesized this protein might play an important role in regulating intracellular *M.tb* survival and replication. To this end, we transiently transfected pcDNA3.1-V5-Rv3722c into RAW264.7 cells and then infected the cells with RFP-H37Ra. Confocal microscopic analysis showed that the number of intracellular bacilli was significantly increased in RAW264.7 cells overexpressing Rv3722c compared to vector control over time (**Figures 2A**). Meanwhile, the number of viable intracellular bacilli per cell was then counted (at least 100 cells per group) after infection at indicated time points (**Figure 2B**).

Considering the low transfection efficiency of RAW264.7 cells, we constructed a RAW264.7 cell line stable overexpressing Rv3722c by using lentiviral infection to facilitate subsequent research (**Figure 2C**). Next, both RAW-Rv3722c and the vector control cell line RAW-Vector were infected with RFP-H37Ra and subjected to bacillus counting based on confocal microscopic analysis. As shown in **Figures 2D, E**, there were more bacilli per cell in RAW-Rv3722c than that of the control cells at day 0, day 1, day 2, and day 3. Furthermore, the CFUs per well were significantly increased in RAW-Rv3722c than that in RAW-Vector cell lines (**Figure 2G**), indicating that Rv3722c promotes the survival of *M.tb* in murine macrophages.

### Knock-Down of TRAF3 Attenuates *M.tb* Proliferation

To test whether Rv3722c promotes *M.tb* survival through TRAF3, we designed and obtained three lentiviruses containing shRNA (shTRAF3-1, shTRAF3-2, or shTRAF3-3) to interfere with endogenous TRAF3 expression in RAW-Rv3722c cell line and performed an intracellular bacillus viable assay. As shown in **Figure 2F**, the qRT-PCR analysis showed that all three shTRAF3 RNAs significantly reduce the expression of endogenous TRAF3. As shTRAF3-1 had the most significant interference effect, it was selected for the subsequent survival assay. shTRAF3-1 and the control scramble RNA were transfected into RAW-Rv3722c cell line respectively and then infected with *M.tb*. Intracellular bacterial viability assay demonstrated that the number of CFUs was significantly increased when overexpression of Rv3722c. Of note, the Rv3722c mediated upregulation of intracellular *M.tb* CFUs was significantly attenuated when TRAF3 was knocked down by shTRAF3 RNA in the RAW-Rv3722c cell line (**Figure 2G**), suggesting that TRAF3 is involved in the Rv3722c promoted *M.tb* proliferation in macrophages.

### *M.tb* Rv3722c Modulates MAPK and NF-κB Pathways *via* TRAF3

**Figure 3:**
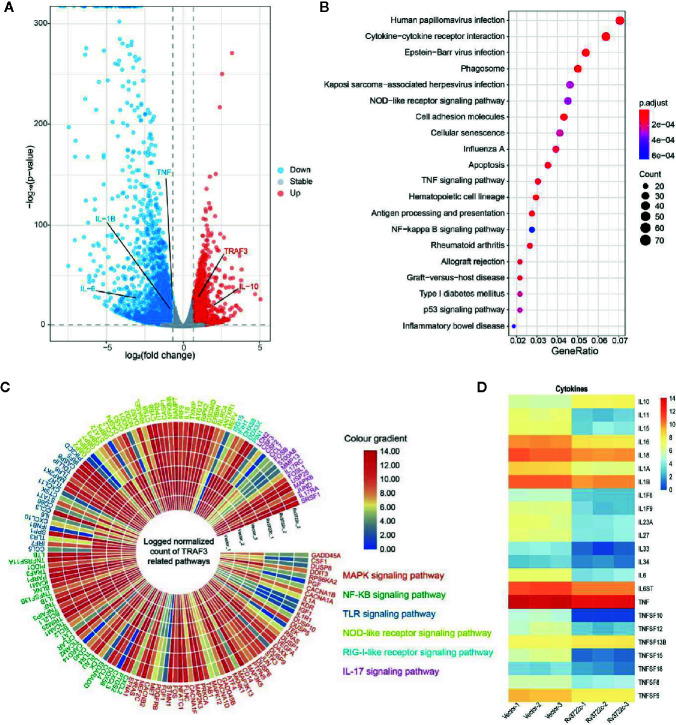
Genes and proteins in different signal pathways are potentially regulated by Rv3722c. **(A)** Volcano plot indicating significant up-regulation (red) and down-regulation (blue) genes after transmission of Rv3722c. **(B)** KEGG enrichment of genes that had significant change overexpression level comparing between RAW-Vector and RAW-Rv3722c cell lines. **(C)** Polarized heatmap showing the normalized gene expression level of TRAF3 related pathways. **(D)** Heatmap showing the normalized count of the expression level of cytokines.

To investigate the host immune responses to Rv3722c, we performed RNA-seq to analyze genes expression profile in RAW264.7 cells upon Rv3722c overexpression. One hundred forty-three significantly differentially expressed genes (DEGs) between RAW-Rv3722c and RAW-Vector cell lines were identified with the threshold of adjusted *p*-value <0.05, log_2_foldchange>1 (**Table S3**). Among these genes, the number of down-regulated genes was much more than that of the up-regulated genes (**Figure 3A**). Comparing to the vector control group, RAW-Rv3722c cell line transcribed a higher level of TRAF3 and IL-10, but a lower level of IL-1β, IL-6, and TNF (**Figure 3A**). KEGG pathway enrichment analysis showed that TRAF3 participated pathways, such as NOD-like receptor, TNF signaling, and NF-κB signaling pathways, were highly enriched (**Figure 3B**), suggesting that Rv3722c may regulate host gene expression by influencing TRAF3 mediated pathways for *M.tb* survival. Furthermore, the gene expression profiles of MAPK signaling, NF-κB signaling, TLR signaling, NOD-like receptor signaling, RIG-I-like receptor signaling, and IL-17 signaling pathways were shown in the polarized heatmap (**Figure 3C**). As these pathways are involved in cytokine expression, we next illustrated cytokine expression from DEGs and generated a heatmap. As shown in **Figure 3D**, RAW-Rv3722c expressed more IL-10 and less IL-1 (both IL-1α and IL-1β), IL-11, IL-15, IL-16, IL-18, and genes in the TNF family than RAW-Vector. These results suggest that Rv3722c might function by affecting inflammation response.

**Figure 4:**
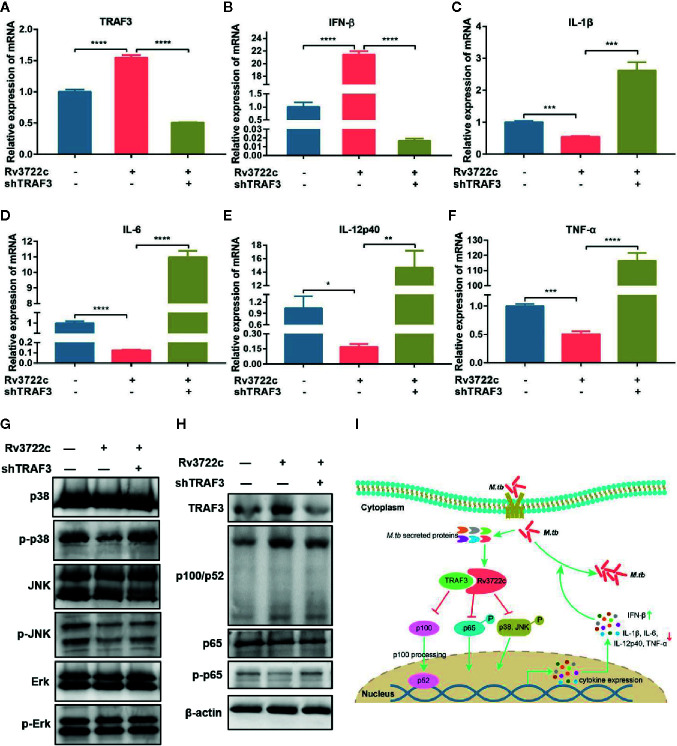
*M.tb* Rv3722c modulates MAPK and NF-κB pathways *via* TRAF3. **(A–F)** qRT-PCR analysis of TRAF3, IFN-β, IL-1β, IL-6, IL-12p40, and TNF-α mRNA expression in RAW-Vector or RAW-Rv3722c cells with or without lentivirus containing shTRAF3-1 gene infection for 48 h. **(G, H)** Western blot analysis of TRAF3, p100/p52, p65, p-p65, p38, p-p38, JNK, p-JNK, Erk, p-Erk expression in RAW-Vector and RAW-Rv3722c cells with or without lentivirus containing shTRAF3 gene infection for 48 h. **(I)** Model of Rv3722c interacted with TRAF3 to regulate host immunity and promote *M.tb* survival. The PAMPs of *M.tb* was recognized by the host PRRs and then parasitized in the host cells. Rv3722c secreted by *M.tb* interacted with TRAF3 in the host cell to inhibit the processing of p100/p52, the phosphorylation of p65, p38, and JNK, promote the secretion of IFN-β, and inhibit the production of IL-1β, IL-6, IL-12p40, and TNF-α, which in turn promoted the proliferation of *M.tb* in host cells. Data shown in (A–F) are mean ± SD of three independent experiments. *P < 0.1; **P < 0.01; ***P < 0.001; ****P < 0.0001; ns, not significant; both by student *t*-test. All experiments were performed in triplicate.

Cytokines are important in the host innate immune response against *M.tb* infection (Cooper and Khader, 2008). We next validated whether Rv3722c modulates the expression of pro-inflammatory and regulatory cytokines by analyzing the expression of mRNAs in macrophages. As shown in **Figures 4A–F**, overexpression of Rv3722c significantly enhanced the expression of TRAF3 and IFN-β, and inhibited the expression of IL-1β, IL-6, IL-12p40, and TNF-α. Notably, knock-down the expression of endogenous TRAF3 could attenuate the Rv3722c induced alteration of the expression of these examined genes by qRT-PCR analysis. To explore the signaling pathways that might be involved in Rv3722c altered cytokine expression, we examined MAPK and NF-κB pathways, which have been described to be involved in the production of numerous inflammatory cytokines (Koul et al., 2004; Jung et al., 2006; Méndez-Samperio, 2010). Therefore, we performed a western blot assay to further examine the proteins and their phosphorylation involved in MAPK and NF-κB pathways. As shown in **Figures 4G, H**, Rv3722c inhibited p65 phosphorylation and p100/p52 processing in NF-κB, and phosphorylation of p38 and JNK in MAPKs pathway, whereas phosphorylation of extracellular signal-regulated kinase (Erk) was not impacted by Rv3722c. Consistent with the cytokine expression experiment, knock-down the endogenous TRAF3 expression attenuated the Rv3722c induced alteration of these examined proteins expression.

Taken together, these results suggest the possibility that Rv3722c hijacked TRAF3 and then influenced NF-κB and MAPK signaling to regulate cytokine expression, which eventually promoted the proliferation of *M.tb* in macrophages (**Figure 4I**).

## Discussion

As an intracellular pathogen, *M.tb* secretes numerous proteins into the cytoplasm of infected macrophages to regulate host innate immunity for its long term persistence. Some of them are identified to suppress host immune responses to promote bacterial survival during infection, such as ESAT6 and a 38 KD glycolipoprotein (Pathak et al., 2007; Wang J. et al., 2015). Rv3722c, an *M.tb* secreted protein, is essential for its *in vitro* growth. Recently, Robert et al. found that Rv3722c is an aspartate aminotransferase and mediates an essential but under-recognized role in metabolism (Jansen et al., 2020). Here, we found that Rv3722c benefited *M.tb* intracellular survival in murine macrophages by modulating TRAF3 mediated cytokine responses.

Accumulating evidence indicates that host-pathogen interaction can facilitate the coevolution of *M.tb* with its host (Gagneux, 2012). Here, we demonstrated that *M.tb* Rv3722c interacted with host TRAF3 by Y2H, BiFC, and Co-IP technologies. TRAF3 is a versatile adaptor protein to regulate signaling complexes downstream of PRRs to activate the type I interferon response and negatively regulate the activation of MAPK and noncanonical NF-κB pathways (Häcker et al., 2011). Our study showed that Rv3722c significantly enhanced the expression of TRAF3 in macrophages. Interestingly, specific knock-down of TRAF3 abrogated Rv3722c induced inhibition of MAPK and NF-κB pathways as well as the production of cytokines in macrophages, suggesting that Rv3722c interacting with TRAF3 to suppress host immune response. In addition, TRAF3 contains four functional domains: RING finger domain, zinc finger domain, coiled coil domain and MATH domain (Zhang et al., 2012). It would be of great interest to further explore which TRAF3 domains involved in Rv3722c binding, whether TRAF3 activity is modulated by *M.tb*, and what is the function of TRAF3 in host defense against *M.tb* infection.

Our RNA-seq analysis revealed that most of the DEGs between RAW-Rv3722c and RAW-vector cell lines were mainly related to MAPK, NF-κB, TLR, and NLR signaling pathways, suggesting that Rv3722c might promote intracellular *M.tb* survival and replication by altering cytokine expression profile. Type I IFN is detrimental for the host to defense intracellular bacteria, such as *Mycobacterium*, *Francisella*, and *Listeria* (Boxx and Cheng, 2016). It has been reported that IFN-β suppresses host anti-bacterial immune responses and facilitates pathogenesis during *M.tb* infection (Donovan et al., 2017; Moreira-Teixeira et al., 2018). Emerging evidence demonstrates that TRAF3 activity can be regulated through different types of ubiquitination. MYD88- and TRIF-dependent K63-linked ubiquitination of TRAF3 activates type I IFN response, whereas cIAP1/2- and TRAF6-mediated K48-linked ubiquitination of TRAF3 induces its proteasomal degradation and induction of proinflammatory cytokines (Tseng et al., 2010). In our study, we found that knock-down of endogenous TRAF3 attenuated the Rv3722c induced up-regulation of IFN-β expression. Hence, we hypothesized that Rv3722c interacted and promoted the synthesis of non-degradative, K63-linked polyubiquitin of TRAF3, which contributed to the activation of IRF3 and subsequent the type I interferon response. TNF-α, IFN-γ, IL-1β, IL-6, IL-12, IL-18, and IL-23 have been reported as defensive cytokines against *M.tb* infection (North and Jung, 2004; Cooper, 2009; Hossain and Norazmi, 2013; Wang J. et al., 2015). Here, we found that knock-down the endogenous TRAF3 abolished the Rv3722c induced decreasing of IL-1β, IL-6, IL-12p40, and TNF-α expression.

We demonstrated that *M.tb* Rv3722c regulated cytokine expression through both MAPK and NF-κB pathways. The phosphorylation and dephosphorylation modifications of proteins are key mechanisms for the regulation of host innate immunity (Fischer, 2013). RNA interference-mediated interferes with TRAF3 degradation is required for TLR-mediated JNK and p38 activation and the production of inflammatory cytokines, such as TNF, IL-6 and IL-12 (Häcker et al., 2011). In this study, we found that Rv3722c might inhibit the MAPK pathway by suppressing the phosphorylation of p38 and JNK through TRAF3. MAPK signaling pathways genes *Gadd45a*, *DUSP1*, *DUSP5*, *DUSP8*, *DUSP10*, and Jun were also found significantly modulated by Rv3722c in macrophages. Whether and how these genes participate in Rv3722c mediated promotion of *M.tb* replication need to be further investigated. Activation of NF-κB is mediated by inflammatory signals or factors involved in the development (Sun, 2017). Here, we found that Rv3722c slightly suppressed the phosphorylation of p65 in the canonical NF-κB pathway and p100/p52 processing in the non-canonical NF-κB pathway in macrophages. Our data implied that Rv3722c might interfere MAPK and NF-κB pathways *via* multiple and complex mechanisms. However, the effects of Rv3722c on the phosphorylation of p38 and JNK seemed to be more obvious than the change in the NF-κB pathway, which hints that Rv3722c might mainly modulate cytokine expression by hampering the MAPK pathway. How Rv3722c participated in inhibiting or interfering with the phosphorylation of examined proteins? A possible mechanism might modulate or interact with a target that is involved in the common regulation of these different proteins.

## Conclusion

Our study revealed that *M.tb* secreted protein Rv3722c interacted with host protein TRAF3 to promote its survival in macrophages through activating the type I interferon response and suppressing MAPK and NF-κB pathways. Our data provided insights into the mechanism of Rv3722c promoting *M.tb* intracellular survival and facilitate further understanding of the mechanisms of *M.tb* secreted proteins in regulating cell immune response.

## Data Availability Statement

RNA-sequencing data have been deposited in Gene Expression Omnibus (GEO) under accession number GSE157419.

## Author Contributions

GC, XC, and YL contributed conception and design of this study. YL, WZ, YX, SD, and DW performed experiments. BY, XJC, and WX performed the protein-protein docking simulation and bioinformatics analysis. YL wrote the first draft of the manuscript, with inputs from all other authors. All the authors discussed the results and commented on the manuscript. All authors contributed to the article and approved the submitted version.

## Funding

This work was financially supported by the National Natural Science Foundation of China (Grant No. 31902240), the China Postdoctoral Science Foundation (Grant No. 2018M640718), the Natural Science Foundation for Youths of Hubei Province of China (Grant No. ZRMS2019000304), the National Key Research and Development Program of China (Grant No. 2017YFD0500303), and the Postdoctoral scientific and technological activities Optimal Funding of Hubei Province of China.

## Conflict of Interest

The authors declare that the research was conducted in the absence of any commercial or financial relationships that could be construed as a potential conflict of interest.
