# Supplementary material for: Rv3722c Promotes Mycobacterium tuberculosis Survival in Macrophages by Interacting With TRAF3
Source: Front Cell Infect Microbiol. 2021 Feb 25;11:627798. doi: 10.3389/fcimb.2021.627798 (PMC7947218; doi:10.3389/fcimb.2021.627798)
Supplement: Supplementary file 1 [file Table_1.docx]

Supplementary Material

# Supplementary Tables

## Table S1. shRNA oligonuleotides sequences

| shNC-F | CCGG CAACAAGATGAAGAGCACCAACTCGAGTTGGTGCTCTTCATCTTGTTG TTTTTG |
| --- | --- |
| shNC-R | AATTCAAAAA CAACAAGATGAAGAGCACCAACTCGAGTTGGTGCTCTTCATCTTGTTG |
| shTRAF3-1F | CCGG GCCAAGAAAGCATCATCAAAGCTCGAGCTTTGATGATGCTTTCTTGGC TTTTTG |
| shTRAF3-1R | AATTCAAAAA GCCAAGAAAGCATCATCAAAGCTCGAGCTTTGATGATGCTTTCTTGGC |
| shTRAF3-2F | CCGG GCAAGAGAGAGATTCTGGCCTCGAG GCCAGAATCTCTCTCTTGC TTTTTG |
| shTRAF3-2R | AATTCAAAAA GCAAGAGAGAGATTCTGGCCTCGAGGCCAGAATCTCTCTCTTGC |
| shTRAF3-3F | CCGG GCCAAGTGCCCATGATCAAACCTCGAGGTTTGATCATGGGCACTTGGC TTTTTG |
| shTRAF3-3R | AATTCAAAAA GCCAAGTGCCCATGATCAAACCTCGAGGTTTGATCATGGGCACTTGGC |

## Table S2. Primers for qRT-PCR

| Primer name | sequence(5'-3') |
| --- | --- |
| M-IFNβ-F | CCTATGGAGATGACGGAGAAGA |
| M-IFNβ-R | AGTGGAGAGCAGTTGAGGACAT |
| M-IL1β-F | AGAAACAGTCCAGCCCATAC |
| M-IL1β-R | CTGGTACATCAGCACCTCAC |
| M-IL6-F | ACAGAAGGAGTGGCTAAGGA |
| M-IL6-R | CGCACTAGGTTTGCCGAGTA |
| M-IL12p40-F | TTGAAAGGCTGGGTATCGGT |
| M-IL12p40-R | GAATTTCTGTGTGGCACTGG |
| M-TNFα-F | TTCTGTCTACTGAACTTCGGGGTGATCGGTCC |
| M-TNFα-R | GTATGAGATAGCAAATCGGCTGACGGTGTGGG |
| M-actin-F | CACTGCCGCATCCTCTTCCTCCC |
| M-actin-R | CAATAGTGATGACCTGGCCGT |

## Table S3. DEGs between RAW-Vector and RAW-Rv3722c cell lines

| Gene Name | Ensembly ID | log_2_FoldChange | padj |
| --- | --- | --- | --- |
| GADD45A | ENSMUSG00000036390 | -4.241612834 | 1.7047E-60 |
| CSF1 | ENSMUSG00000014599 | -3.998076545 | 3.92002E-28 |
| DUSP8 | ENSMUSG00000037887 | -3.83475808 | 1.08964E-37 |
| DDIT3 | ENSMUSG00000025408 | -3.655198923 | 2.8209E-222 |
| RPS6KA2 | ENSMUSG00000023809 | -3.214127036 | 2.34914E-49 |
| PGF | ENSMUSG00000004791 | -3.113559795 | 3.21835E-09 |
| CACNA1B | ENSMUSG00000004113 | -2.306992911 | 4.10903E-23 |
| CACNA1A | ENSMUSG00000034656 | -2.302093156 | 1.79466E-92 |
| IL1A | ENSMUSG00000027399 | -2.27578299 | 2.68619E-48 |
| KDR | ENSMUSG00000062960 | -2.260809104 | 5.51747E-05 |
| IGF1 | ENSMUSG00000020053 | -2.121425345 | 0.000143323 |
| IL1R1 | ENSMUSG00000026072 | -2.047127816 | 0.001007576 |
| DUSP10 | ENSMUSG00000039384 | -2.045141087 | 9.39372E-24 |
| DUSP5 | ENSMUSG00000034765 | -1.909555105 | 6.6134E-110 |
| TEK | ENSMUSG00000006386 | -1.821012117 | 0.004688135 |
| RRAS | ENSMUSG00000038387 | -1.749783137 | 1.71622E-20 |
| DUSP1 | ENSMUSG00000024190 | -1.722606716 | 9.79621E-51 |
| VEGFA | ENSMUSG00000023951 | -1.641705805 | 2.13157E-91 |
| DAXX | ENSMUSG00000002307 | -1.550974842 | 2.63165E-75 |
| DUSP9 | ENSMUSG00000031383 | -1.536295253 | 0.000912209 |
| STK3 | ENSMUSG00000022329 | -1.495372707 | 1.31799E-31 |
| JUN | ENSMUSG00000052684 | -1.472907911 | 1.2219E-47 |
| DUSP6 | ENSMUSG00000019960 | -1.332855174 | 3.29937E-36 |
| PTPN5 | ENSMUSG00000030854 | -1.310073159 | 9.79639E-29 |
| MAP3K5 | ENSMUSG00000071369 | -1.266426913 | 1.14595E-34 |
| CD14 | ENSMUSG00000051439 | -1.225414792 | 2.95087E-76 |
| MAP3K13 | ENSMUSG00000033618 | -1.147419257 | 0.001886331 |
| MKNK1 | ENSMUSG00000028708 | -1.130980991 | 6.97253E-41 |
| ATF4 | ENSMUSG00000042406 | -1.102363953 | 5.14288E-27 |
| GADD45B | ENSMUSG00000015312 | -1.046946152 | 3.40577E-18 |
| CACNA1D | ENSMUSG00000015968 | -1.026625716 | 0.000247454 |
| MAPK12 | ENSMUSG00000022610 | -1.015799681 | 5.07051E-06 |
| TAB1 | ENSMUSG00000022414 | 1.012735946 | 2.44323E-15 |
| PRKCA | ENSMUSG00000050965 | 1.027640133 | 1.41364E-40 |
| MAP2K3 | ENSMUSG00000018932 | 1.029547352 | 1.05319E-42 |
| CACNA1F | ENSMUSG00000031142 | 1.063281222 | 1.17444E-08 |
| FLNC | ENSMUSG00000068699 | 1.066781896 | 4.35574E-19 |
| NFATC1 | ENSMUSG00000033016 | 1.2890333 | 3.52694E-90 |
| FOS | ENSMUSG00000021250 | 1.314247682 | 2.19529E-42 |
| STMN1 | ENSMUSG00000028832 | 1.346568951 | 3.14384E-27 |
| FGF1 | ENSMUSG00000036585 | 1.601419568 | 0.020011831 |
| PDGFRB | ENSMUSG00000024620 | 1.615012036 | 4.23893E-05 |
| MET | ENSMUSG00000009376 | 2.09742405 | 4.32275E-11 |
| CACNB2 | ENSMUSG00000057914 | 3.680600746 | 1.223E-70 |
| MEF2C | ENSMUSG00000005583 | 0.72240863 | 3.18522E-16 |
| HRAS | ENSMUSG00000025499 | 0.706051042 | 3.84867E-07 |
| EFNA1 | ENSMUSG00000027954 | 0.981309686 | 0.005108449 |
| CXCL2 | ENSMUSG00000058427 | -5.321525683 | 0 |
| PTGS2 | ENSMUSG00000032487 | -3.896603739 | 0 |
| CXCL3 | ENSMUSG00000029379 | -3.737069251 | 5.20928E-11 |
| DDX58 | ENSMUSG00000040296 | -3.556842675 | 1.5806E-243 |
| CCL4 | ENSMUSG00000018930 | -2.844419264 | 1.1845E-208 |
| EDARADD | ENSMUSG00000095105 | -2.458760416 | 7.40217E-07 |
| LCK | ENSMUSG00000000409 | -2.018006972 | 2.41126E-57 |
| PLAU | ENSMUSG00000021822 | -1.887453586 | 8.24511E-88 |
| TLR4 | ENSMUSG00000039005 | -1.539356531 | 6.05315E-59 |
| CD40 | ENSMUSG00000017652 | -1.498373446 | 6.55073E-32 |
| CARD14 | ENSMUSG00000013483 | -1.463273538 | 0.034050815 |
| TICAM2 | ENSMUSG00000056130 | -1.34075498 | 8.71243E-27 |
| CFLAR | ENSMUSG00000026031 | -1.29386144 | 3.33393E-84 |
| LAT | ENSMUSG00000030742 | -1.214823578 | 0.00108977 |
| BCL2 | ENSMUSG00000057329 | -1.125875986 | 3.94216E-18 |
| TICAM1 | ENSMUSG00000047123 | -1.095862152 | 2.37827E-31 |
| TRIM25 | ENSMUSG00000000275 | -1.071254636 | 4.0559E-36 |
| BCL2L1 | ENSMUSG00000007659 | -1.06069961 | 5.58214E-42 |
| TNFAIP3 | ENSMUSG00000019850 | -1.011948511 | 3.79466E-22 |
| TNF | ENSMUSG00000024401 | -0.793711661 | 9.2981E-26 |
| IL1B | ENSMUSG00000027398 | -0.785912309 | 3.70952E-15 |
| TNFSF13B | ENSMUSG00000031497 | 0.683048011 | 4.37264E-06 |
| BLNK | ENSMUSG00000061132 | 0.741533105 | 1.3981E-12 |
| ICAM1 | ENSMUSG00000037405 | 0.743452886 | 1.38537E-26 |
| PARP1 | ENSMUSG00000026496 | 0.785638304 | 4.45816E-36 |
| TRAF3 | ENSMUSG00000021277 | 0.897388976 | 1.00692E-27 |
| PIDD1 | ENSMUSG00000025507 | 1.088404266 | 8.82521E-21 |
| TNFRSF11A | ENSMUSG00000026321 | 1.128842919 | 4.47905E-46 |
| LTB | ENSMUSG00000024399 | 2.130691178 | 1.1442E-151 |
| CCL5 | ENSMUSG00000035042 | -5.765999169 | 3.0238E-116 |
| IRF7 | ENSMUSG00000025498 | -5.144269289 | 0 |
| TLR3 | ENSMUSG00000031639 | -4.108763019 | 3.6899E-215 |
| SPP1 | ENSMUSG00000029304 | -3.855370612 | 0 |
| IFNB1 | ENSMUSG00000048806 | -3.545829982 | 7.79575E-11 |
| CXCL10 | ENSMUSG00000034855 | -3.109044343 | 1.2002E-138 |
| IL6 | ENSMUSG00000025746 | -2.92750469 | 4.48825E-27 |
| CCL3 | ENSMUSG00000000982 | -2.8546239 | 9.9979E-173 |
| CD86 | ENSMUSG00000022901 | -2.782245466 | 1.80292E-21 |
| STAT1 | ENSMUSG00000026104 | -2.067371065 | 1.7756E-114 |
| CTSK | ENSMUSG00000028111 | -2.003025395 | 2.90659E-17 |
| CXCL11 | ENSMUSG00000060183 | -1.715653081 | 0.002407354 |
| TLR7 | ENSMUSG00000044583 | -1.128953713 | 3.72589E-35 |
| MAP2K1 | ENSMUSG00000004936 | -0.985660933 | 5.74696E-28 |
| TLR6 | ENSMUSG00000051498 | -0.767610963 | 1.98123E-12 |
| TOLLIP | ENSMUSG00000025139 | -0.762095619 | 7.18675E-17 |
| CASP8 | ENSMUSG00000026029 | -0.694975989 | 1.78398E-18 |
| IRF5 | ENSMUSG00000029771 | 0.738711484 | 1.43541E-25 |
| PIK3CD | ENSMUSG00000039936 | 1.01649998 | 3.39816E-33 |
| GBP2 | ENSMUSG00000028270 | -5.575889652 | 8.67284E-56 |
| GBP4 | ENSMUSG00000079363 | -4.38279794 | 6.50127E-15 |
| GBP7 | ENSMUSG00000040253 | -4.252140444 | 3.2885E-189 |
| GBP3 | ENSMUSG00000028268 | -3.956935584 | 4.0985E-281 |
| STAT2 | ENSMUSG00000040033 | -3.800007831 | 0 |
| GBP5 | ENSMUSG00000105504 | -3.704002244 | 2.09101E-16 |
| CCL2 | ENSMUSG00000035385 | -3.656977378 | 1.5191E-116 |
| CASP4 | ENSMUSG00000033538 | -2.731378027 | 3.5772E-205 |
| OAS3 | ENSMUSG00000032661 | -2.345116042 | 7.556E-95 |
| NOD1 | ENSMUSG00000038058 | -2.107110779 | 2.42514E-05 |
| NOD2 | ENSMUSG00000055994 | -2.078902197 | 2.87257E-44 |
| OAS2 | ENSMUSG00000032690 | -1.921231823 | 3.06454E-73 |
| RNASEL | ENSMUSG00000066800 | -1.887210326 | 6.69601E-47 |
| CTSB | ENSMUSG00000021939 | -1.738345583 | 4.7862E-122 |
| CYBB | ENSMUSG00000015340 | -1.518742143 | 1.8938E-105 |
| ITPR2 | ENSMUSG00000030287 | -1.469175659 | 2.60464E-61 |
| CASP1 | ENSMUSG00000025888 | -1.305725453 | 2.71923E-38 |
| NFKBIB | ENSMUSG00000030595 | -1.292279651 | 6.21148E-25 |
| NAMPT | ENSMUSG00000020572 | -1.251249802 | 1.25711E-41 |
| IRF9 | ENSMUSG00000002325 | -1.191279337 | 6.73562E-47 |
| IL18 | ENSMUSG00000039217 | -0.960448632 | 1.60808E-16 |
| TXNIP | ENSMUSG00000038393 | -0.917534252 | 7.89801E-51 |
| ATG12 | ENSMUSG00000032905 | -0.880369194 | 4.43503E-14 |
| RIPK2 | ENSMUSG00000041135 | -0.782924176 | 2.23382E-18 |
| GSDMD | ENSMUSG00000022575 | -0.775211849 | 5.39369E-24 |
| ERBIN | ENSMUSG00000021709 | -0.678167544 | 4.38165E-19 |
| NLRC4 | ENSMUSG00000039193 | 1.260476985 | 3.36143E-19 |
| ANTXR1 | ENSMUSG00000033420 | 1.340805113 | 1.86452E-14 |
| MEFV | ENSMUSG00000022534 | 1.572637312 | 0.020114543 |
| ISG15 | ENSMUSG00000035692 | -6.439448429 | 0 |
| IFIH1 | ENSMUSG00000026896 | -4.187586851 | 0 |
| DHX58 | ENSMUSG00000017830 | -3.271443091 | 5.65E-173 |
| DDX3X | ENSMUSG00000000787 | 0.718527536 | 1.65297E-34 |
| PIN1 | ENSMUSG00000032171 | 0.740254085 | 1.54424E-17 |
| LCN2 | ENSMUSG00000026822 | -7.450485714 | 3.3788E-198 |
| CSF3 | ENSMUSG00000038067 | -5.589121108 | 0 |
| CSF2 | ENSMUSG00000018916 | -4.275641472 | 1.53797E-17 |
| CCL7 | ENSMUSG00000035373 | -2.70158509 | 2.24128E-32 |
| FOSB | ENSMUSG00000003545 | -2.21585276 | 2.38319E-12 |
| S100A8 | ENSMUSG00000056054 | -1.926247229 | 2.12375E-05 |
| MMP13 | ENSMUSG00000050578 | -1.5875194 | 0.000544146 |
| IL17RC | ENSMUSG00000030281 | -1.398737066 | 1.08019E-20 |
| FOSL1 | ENSMUSG00000024912 | -0.919693278 | 1.40624E-21 |
| USP25 | ENSMUSG00000022867 | -0.884438799 | 2.91914E-24 |
| MAPK6 | ENSMUSG00000042688 | -0.754766689 | 1.65024E-33 |
| JUND | ENSMUSG00000071076 | -0.6957618 | 7.66383E-05 |
| IL17RA | ENSMUSG00000002897 | 0.712638142 | 1.10234E-22 |
| SRSF1 | ENSMUSG00000018379 | 0.88413289 | 3.7906E-22 |
